# Exhaled Nitric Oxide is Not a Biomarker for Pulmonary Tuberculosis

**DOI:** 10.4269/ajtmh.17-0425

**Published:** 2018-04-30

**Authors:** José W. López, Maria-Cristina I. Loader, Daniel Smith, Daniel Pastorius, Marjory Bravard, Luz Caviedes, Karina M. Romero, Taryn Clark, William Checkley, Eduardo Ticona, Jon S. Friedland, Robert H. Gilman

**Affiliations:** 1 Laboratorio de Investigación en Enfermedades Infecciosas, Laboratorio de Investigación y Desarrollo, Facultad de Ciencias y Filosofía, Universidad Peruana Cayetano Heredia, Lima, Peru;; 2 Instituto Nacional de Salud del Niño, San Borja, Lima, Peru;; 3 Section of Infectious Diseases and Immunity, Wellcome Centre for Global Health, Imperial College London, London, United Kingdom;; 4 Asociación Benéfica PRISMA, Lima, Peru;; 5 Department of General Medicine, Massachusetts General Hospital, Boston, Massachusetts;; 6 Section of Emergency Medicine, Department of Medicine, Louisiana State University Health Sciences Center, New Orleans, Louisiana;; 7 Department of International Health, Bloomberg School of Public Health, Johns Hopkins University, Baltimore, Maryland;; 8 Division of Pulmonary and Critical Care, School of Medicine, Johns Hopkins University, Baltimore, Maryland;; 9 CRONICAS Center in Chronic Diseases, Universidad Peruana Cayetano Heredia, Lima, Peru;; 10 Hospital Nacional Dos de Mayo, Lima, Peru;; 11 Facultad de Medicina de la Universidad Nacional Mayor de San Marcos, Lima, Peru

## Abstract

To reduce transmission of tuberculosis (TB) in resource-limited countries where TB remains a major cause of mortality, novel diagnostic tools are urgently needed. We evaluated the fractional concentration of exhaled nitric oxide (FeNO) as an easily measured, noninvasive potential biomarker for diagnosis and monitoring of treatment response in participants with pulmonary TB including multidrug resistant–TB in Lima, Peru. In a longitudinal study however, we found no differences in baseline median FeNO levels between 38 TB participants and 93 age-matched controls (13 parts per billion [ppb] [interquartile range (IQR) = 8–26] versus 15 ppb [IQR = 12–24]), and there was no change over 60 days of treatment (15 ppb [IQR = 10–19] at day 60). Taking this and previous evidence together, we conclude FeNO is not of value in either the diagnosis of pulmonary TB or as a marker of treatment response.

New clinical tools are urgently needed to reduce the transmission of tuberculosis (TB) and multi-drug resistant (MDR)–TB in resource-limited countries where TB remains a major cause of mortality.^1^ In 2015, there were an estimated 10.4 million incident cases with 1.4 million deaths worldwide.^2^ In Peru, the TB incidence in 2015 was 119 per 100,000 people, with the highest MDR-TB rate in South America.^2^ In mice, nitric oxide (NO) is important in TB killing by murine mononuclear phagocytes and, in latent murine infection, NO synthase (NOS) inhibitors drive dissemination of *Mycobacterium tuberculosis*.^3^ The importance of NO in human TB, however, is controversial. Inducible NOS (iNOS) activity in human macrophages may be upregulated by TB, but early mycobacteriostatic activity of alveolar macrophages infected with *Mycobacterium tuberculosis* has been shown to be NO independent.^4^ Conversely, supplementation of arginine, a substrate for NO production, in participants undergoing TB treatment improved clinical outcomes, including sputum conversion and time to cessation of symptoms.^5^ Fractional concentration of exhaled NO (FeNO) is an easily measured noninvasive test, standardized to guide diagnosis and assess the likelihood of treatment response to corticosteroids in atopy and asthma.^6,7^ This study evaluated the diagnostic utility of FeNO in smear-positive TB participants and longitudinal change in FeNO during treatment as a potential marker of treatment failure due to drug resistance.

Tuberculosis participants older than 18 years of age, nonsmokers, not pregnant, and with no history/family history of atopy or asthma were recruited at Hospital Nacional Dos de Mayo, Lima, Peru. Pulmonary TB was diagnosed by auramine microscopy for acid-fast bacilli and/or by culture using microscopic observation drug susceptibility assay.^8^ Treatment followed national TB guidelines. A random selection of 120 asymptomatic nonsmoking adults was contemporaneously recruited as controls from a socioeconomically similar neighborhood in Lima. All participants completed a standard questionnaire documenting clinical and demographic characteristics, TB risk factors, and potential factors affecting FeNO levels such as food and beverages consumed, medications, and comorbidities.^9^ All participants gave written informed consent. The study was approved by the institutional review boards of Hospital Nacional Dos de Mayo and Asociación Benéfica PRISMA, Lima, Peru.

Fractional concentrations of exhaled nitric oxide were measured using a portable NO analyzer (NIOX MINO airway inflammation monitor; Aerocrine, Solna, Sweden). Device measurements agreed with measurements provided by a stationary analyzer, as per American Thoracic Society recommendations.^9^ For the TB group, measurements were taken on days 0, 7, 14, 21, 30, and 60 after initiation of treatment (day 0). Controls underwent a single FeNO measurement and tuberculin skin test (TST), categorized as negative using < 10 mm induration cutoff. Breathing parameters required exhalation pressures of 10–20 cm H_2_O and a fixed flow rate of 50 ± 5 mL/s maintained for 6 seconds. The accuracy range for the device is ±5 parts per billion (ppb) for values < 50 ppb. Measurements were taken between 9:00 and 12:00 am in fasting subjects.

Data analysis was performed using Stata/SE 12.0 (Stata Corp., College Station, TX). After double data entry, a univariate analysis of clinical and demographic characteristics was performed. Data were analyzed by using the Wilcoxon rank-sum test, Kruskal–Wallis test, χ^2^ test, or *t* test, as appropriate. The FeNO values were normalized using logarithmic transformations. Linear and multiple regression analyses assessed relationships between FeNO levels and clinical data. Longitudinal analysis of FeNO used one-way analysis of variance for repeated measurements.

Of the 38 TB participants recruited, 7 (18.4%) were drug resistant: three mono-resistant (7.9%) and four MDR (10.5%). Twenty-one participants completed the longitudinal study and a further 17 achieved a minimum of three measurements. The control group included 93 subjects following exclusion of 22 participants with comorbidities, two unable to complete exhaled NO measurement, and three with recent respiratory tract infection.

Participant demographics are summarized in Table 1. There was a greater proportion of male TB than control subjects (78.9% versus 52.7%, *P* = 0.005). Weight (55.8 ± 9.4 kg versus 65.8 ± 12.8 kg) and body mass index (BMI) (21.8 ± 3.3 kg/m^2^ versus 26.8 ± 5.2 kg/m^2^) but not height were significantly different between TB participants and controls. Three TB participants were human immunodeficiency virus (HIV) positive, although their BMI was not significantly different from that of HIV-negative TB participants. The BMI of MDR participants was not significantly different from that of drug-sensitive participants (20.3 ± 2.7 kg/m^2^ versus 21.8 ± 3.3 kg/m^2^). Tuberculosis participants and controls did not differ in terms of food intake or other FeNO influencing factors.

**Table 1:** Baseline characteristics and comparison of FeNO levels, cases vs. controls

	TB participants*	Community controls*	*P* value
	*N* = 38	*N* = 93	
Gender, male, %	30 (78.9)	49 (52.7)	0.005
Age, years	36.1 (17.1)	39.9 (15.3)	NS
Weight, kg	55.8 (9.4)	65.8 (12.8)	0.001
Height, cm	160.1 (8.8)	154.8 (18.8)	NS
BMI	21.8 (3.3)	26.8 (5.2)	0.001
Previous episode of TB, %	13 (34.2)	0.0	–
Household contact with active TB, %	6 (16.2)	0.0	–
Family member undergoing TB treatment, %	3 (7.9)	1 (1.08)	0.027
Weekly expenditure on food, USD$ (median, IQR)	36 (29–72)	53 (40–67)	0.027
Number of rooms in home (median, IQR)	2 (1–4)	3 (2–4)	NS
Number of family members in home (median, IQR)	4 (1–9)	5 (2–10)	NS
FeNO, ppb (median, IQR)	13 (8–26)	15 (12–24)	0.23

BMI = body mass index; FeNO = fractional concentration of exhaled nitric oxide; IQR = interquartile range; NS = not significant; ppb = parts per billion; TB = tuberculosis.

* All data are mean (standard deviation), unless otherwise stated.

Median FeNO levels (Table 1) were 13 ppb in TB participants (interquartile range [IQR] = 8–26) and 15 ppb in healthy controls (IQR = 12–24), which were not significantly different. The FeNO levels between study groups were not significantly affected by age or BMI. Median FeNO levels in TST positive controls were 18 ppb (IQR = 13–27), TST negative controls 13 ppb (IQR = 11–20), and TB cases 13 ppb (IQR = 8–26) and were not significantly different between groups. Multivariate analysis demonstrated no significant association between diagnosis of TB and FeNO levels after adjusting for age, gender, height, BMI, and HIV status (unadjusted odds ratio (OR): 1.15; 95% confidence interval [CI]: 0.92–1.43; adjusted OR: 1.24; 95% CI: 0.96–1.59). In the 38 TB participants followed longitudinally for up to 60 days, there were no significant changes in FeNO levels in pulmonary TB participants during treatment (Figure 1).

**Figure 1.**
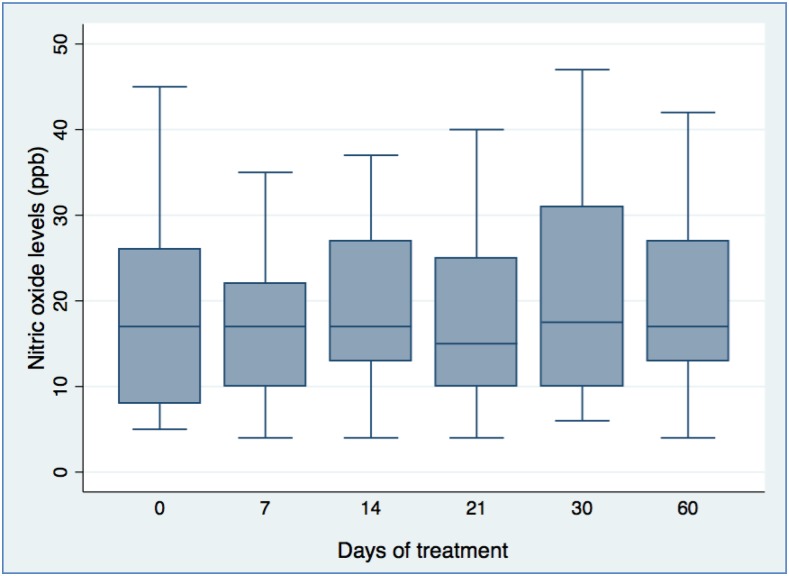
Longitudinal FeNO measurements of tuberculosis (TB) cases. Thirty-eight TB participants were followed longitudinally over 60 days. Twenty-one participants completed six FeNO measurements and a further 17 participants achieved three measurements during this study period. No significant changes in FeNO levels in pulmonary TB participants during treatment were observed during this time. FeNO = fractional concentration of exhaled nitric oxide; ppb = parts per billion. This figure appears in color at www.ajtmh.org.

This study found no differences in median FeNO levels between TB participants and age-matched controls with no change over 2 months of treatment, consistent with findings from a recent study in Korea.^10^ Earlier human FeNO studies had shown conflicting results. The largest human trial to date in Indonesia found lower mean FeNO levels in TB participants, although data overlapped considerably with those in healthy controls, and similar findings were observed in an Ethiopian study which included HIV-positive participants.^11,12^ In contrast, a study in Taiwan reported increased FeNO in active TB patients, postulating increased lung macrophage activity and upregulation of iNOS.^13^

We observed a median FeNO value of 15 ppb (IQR = 12–24) in healthy control subjects, similar to values reported in Vietnamese construction workers (15 ppb, IQR = 12–19) and healthy controls in the Indonesian study (16.6 ppb; 95% CI: 14.2–19.5), but higher than that in controls in the Taiwanese study (6.5 ± 0.9 ppb) and lower than mean values described in Korean controls (27.06 ± 10.8 ppb).^10,11,13,14^ The FeNO level variability in healthy controls was furthermore demonstrated in the Vietnam study: although FeNO was greater in TB participants compared with a subgroup of healthy hospital workers, they were not significantly different when compared with healthy construction workers.^14^ Participants with poor nutritional status were excluded from the Taiwanese study because of concerns regarding the effect on immunity.^13^ Because TB patients are typically wasted and we assessed FeNO as a practical field tool, we made no such exclusions. In addition, unlike the present study, factors known to modulate FeNO such as diet were not examined in the analysis of the Indonesian and Ethiopian populations.^11,12^ Diverse factors influence FeNO generation including consumption of certain foods, smoking, and other comorbidities, making this a challenging test to control for.^9^ In all these divergent human studies, FeNO levels in both TB participants and healthy controls fall below the suggested upper limit of FeNO in never-smoking adults, further calling into question its use as a clinical tool for TB.^15^ One possible limitation of this study is that the analyzer used measures large airway not intracellular alveolar NO concentrations. However, upregulated iNOS expression by alveolar macrophages is associated with increased NO concentration in exhaled breath.^13^ Fractional concentration of exhaled nitric oxide correlates with sputum eosinophilia and Th2 cytokines including IL-5 and IL-13, and has been used as a measure of type 2 airway inflammation.^16,17^ As a Th1 response predominates in TB, this may explain why we found no difference in FeNO levels between TB cases and controls.^18^ Murine TB studies possibly have shown a greater role for FeNO because of different pathogeneses—mice do not show features of postprimary TB, notably caseation. It might, therefore, be useful to explore FeNO in primary TB only.

Our second objective was to determine if FeNO changes occur over time during TB treatment. This might predict failure to respond to antimycobacterial therapy, providing an early warning of drug resistance. We observed that FeNO levels did not respond to TB treatment. This is in keeping with the lack of initial difference between TB and control groups, and with pediatric FeNO data showing stable levels over 1.5 years.^7^ The recent longitudinal study in Korean TB participants also demonstrated no significant change following 8 weeks of treatment.^10^ Similarly, only a very small change was observed in mean FeNO levels at treatment completion in the Indonesian study, 10.7–15.1 ppb, which falls within the accuracy range for the portable NO analyzer device used (±5 ppb for values < 50 ppb). Although, because of the limitation of our small sample size and low statistical power, we cannot exclude a small effect that might be observed in a larger study, this would not be clinically useful.

Taking all the evidence together, our data are consistent with the concept that FeNO is not of value in the direct diagnosis of pulmonary TB, or as a marker of treatment response, and suggest development of other diagnostic tools should be pursued in its place.
